# Association Between Dietary Intake of Omega‐3 Fatty Acids and Serum Testosterone in Adult Males: Results From NHANES 2011–2016 and 2021–2023

**DOI:** 10.1002/fsn3.71062

**Published:** 2025-10-08

**Authors:** Liang Su, Si‐zheng Zhang, Hao‐yang Cheng, Qi Zhang, Zheng‐yang Zhou, Jie Wu, Yong‐zheng Jiao

**Affiliations:** ^1^ Guang'anmen Hospital China Academy of Chinese Medical Sciences Beijing China; ^2^ Beijing University of Chinese Medicine Beijing China; ^3^ Eye Hospital China Academy of Chinese Medical Sciences Beijing China

**Keywords:** male adults, omega‐3, serum testosterone, The National Health and Nutrition Examination Survey

## Abstract

Mounting research reports that omega‐3 fatty acids (Omega‐3) may help improve health, but research on Omega‐3 and male testosterone is limited. Our study included males aged ≥ 18 years in the 2011–2016 and 2021–2023 National Health and Nutrition Examination Survey. Multivariable regression analysis and generalized additive models and smooth curve fitting were used to explore the association between dietary intake of Omega‐3 (including total Omega‐3, docosahexaenoic acid [DHA], eicosapentaenoic acid [EPA], alpha linolenic acid [ALA], docosapentaenoic acid [DPA], and stearidonic acid [SDA]) and male testosterone. Subgroup analyses were performed according to body mass index (BMI) type. 8686 participants were included. Multivariable linear regression found that only DPA exhibited a significant positive association with serum testosterone, and generalized additive models and smooth curve fitting also showed a linear relationship. In subgroup analyses, total Omega‐3 and ALA were significantly positively associated with serum testosterone in the BMI < 25 group. DHA, EPA, DPA, and SDA had statistically significant positive associations in the BMI ≥ 30 group. Besides, SDA showed a statistically significant negative association in the BMI 25–30 group. Our findings indicate that a significant positive association between Omega‐3 intake and male testosterone levels was observed only in the presence of specific Omega‐3 types (DPA) and BMI categories (BMI < 25 kg/m^2^ and BMI ≥ 30 kg/m^2^). Furthermore, cross‐sectional studies cannot establish causality. Therefore, these results require validation through interventional studies.

## Graphical abstract

The DPA intake showed a statistically significant positive association with serum testosterone levels in adult males. In the normal weight participants (BMI < 25 kg/m^2^), total Omega‐3 and ALA showed significant positive associations with testosterone levels. In the obese participants (BMI ≥ 30 kg/m^2^), SDA, EPA, DPA, and DHA showed significant positive associations with testosterone levels. Figure that represents the scope of the paper.

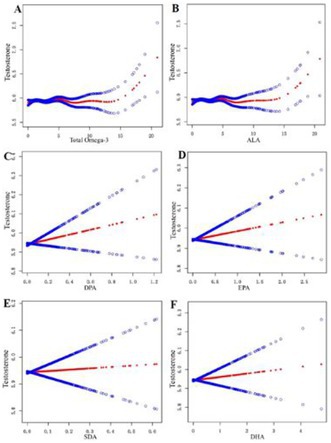

**Abbreviations**
ALA: Alpha‐Linolenic Acid
BMI: Body Mass Index
DHA: Docosahexaenoic Acid
DPA: Docosapentaenoic Acid
EPA: Eicosa Pentaenoic Acid
HPG: Hypothalamus–Pituitary–Gonadal Axis
NHANES: National Health And Nutrition Examination Survey
Omega‐3: Omega‐3 Fatty Acids
SDA: Stearidonic Acid

## 1 Background

Testosterone is an essential male sex hormone that plays a critical role in muscle mass, bone strength, hair growth, sexual functioning, and male fertility (Halpern and Brannigan 2019; Zitzmann et al. 2025). Accumulating evidence suggests that low testosterone levels are linked to widespread chronic diseases, including metabolic syndrome, type 2 diabetes, and cardiovascular disease (Miner et al. 2025; Rao et al. 2013). Adverse lifestyle factors have been shown to significantly correlate with decreased testosterone levels in men (Allen and Walter 2019; Midttun et al. 2024). Notably, diet is now recognized as an important component of lifestyle and a modifiable risk factor for the development of chronic diseases (Cases et al. 2015). Consequently, the relationship between diet and testosterone levels has attracted a lot of attention.

Omega‐3 fatty acids (Omega‐3) are a class of essential polyunsaturated fatty acids that play a significant role in human metabolism, primarily including the following types: docosahexaenoic acid (DHA), eicosapentaenoic acid (EPA), alpha‐linolenic acid (ALA), docosapentaenoic acid (DPA), and stearidonic acid (SDA) (Swanson et al. 2012; Wang et al. 2025). Dietary intake of more Omega‐3 has been reported to protect against inflammation, cardiovascular disease, cognitive decline, cancer, and other chronic diseases (Li et al. 2025; Munhoz et al. 2025; Tseng et al. 2025). Animal studies have demonstrated that dietary Omega‐3 altered the lipid composition of rat testicular plasma membranes, thereby altering testicular mesenchymal cell reactivity (Sebokova et al. 1990). Another animal study showed that Omega‐3 fatty acids improved sexual and erectile function in rats by upregulating NO/cGMP signaling and steroidogenic enzyme activity (Odetayo and Olayaki 2023). Notably, clinical studies examining the relationship between Omega‐3 and testosterone remain insufficiently explored. Fish is rich in Omega‐3; according to a cross‐sectional study conducted in Japan, increased fish intake was significantly associated with elevated serum testosterone levels in older men (Ito et al. 2024). However, a separate double‐blind, placebo‐controlled supplementation study reported that Omega‐3 supplementation did not significantly influence serum testosterone levels in older men with a history of myocardial infarction (Giltay et al. 2012). Thus, the relationship between Omega‐3 intake and serum testosterone levels is currently unclear. Furthermore, neither study explores the relationship between various Omega‐3 and testosterone in detail. A secondary exploratory analysis of a small sample of randomized controlled trials in men indicated that changes in testosterone levels were positively correlated with variations in EPA and DHA, and that DHA‐enriched fish oil supplements increased testosterone levels in overweight and obese men (Abbott et al. 2020). This study suggests that the type of Omega‐3 and obesity may be important factors in the relationship between Omega‐3 and testosterone. Moreover, unhealthy dietary habits have contributed to the global rise in obesity, which has also been associated with a significant decline in male testosterone levels (Fernández‐García et al. 2022; Wang et al. 2018).

Therefore, we utilized data from the National Health and Nutrition Examination Survey (NHANES) to examine the association between dietary Omega‐3 intake and serum testosterone levels in adult males. In addition, given that obesity is a key contributor to testosterone decline and considering the rapid global rise in obesity prevalence (Grossmann 2018), we conducted stratified analyses based on obesity status.

## 2 Methods

### 2.1 Study Population

Data were obtained from NHANES, a serial cross‐sectional survey that collects information on the nutritional and health status of the noninstitutionalized U.S. population. This nationwide survey provides comprehensive data through questionnaires, physical examinations, and laboratory tests. The investigation protocol was approved by the National Center for Health Statistics Ethics Review Board, and each participant provided written informed consent (Kang et al. 2025).

Four cycles of NHANES data (2011–2012, 2013–2014, 2015–2016, and 2021–2023) were used for this analysis, as only these survey cycles included serum testosterone. We included men aged 18 years or older with available information on serum testosterone, DHA, EPA, ALA, DPA, and SDA. Participants with missing data were excluded from the analysis. Additionally, to address potential under‐ or over‐nutrition, we excluded participants with extreme energy intake (≤ 500 or ≥ 8000 kcal) (Liu et al. 2022). Moreover, participants with missing covariates were also excluded.

### 2.2 Exposure and Outcome Definitions

In this study, the primary exposure variable was Omega‐3, including both total Omega‐3 intake and its individual components: DHA, EPA, ALA, DPA, and SDA. Intake data were obtained from the dietary component of NHANES, which utilizes 24‐h dietary recall interviews. Dietary data were collected through an initial in‐person interview conducted at the Mobile Examination Center (MEC), followed by a second recall conducted by telephone 3–10 days later (Ma et al. 2024). Given the potential for measurement error and recall bias—particularly in the second, remotely administered dietary recall—we limited our analysis to data obtained from the first, face‐to‐face dietary interview (Zhang et al. 2023). The outcome variable was serum testosterone, which was measured using isotope dilution liquid chromatography–tandem mass spectrometry, a method with high specificity and sensitivity (Su et al. 2022).

### 2.3 Covariates

Considering that age, body mass index (BMI), race, diabetes, hypertension, and cholesterol may affect male serum testosterone and Omega‐3, these variables were included in multivariable models (Hernández‐Pérez et al. 2024; Su et al. 2022). In the subgroup analysis stratified by BMI, we categorized participants into three levels (normal: < 25 kg/m^2^; overweight: 25–30 kg/m^2^; obesity: ≥ 30 kg/m^2^) (Daher et al. 2025). Diabetes, hypertension, and high cholesterol were confirmed by self‐reported clinician diagnosis.

### 2.4 Statistical Analysis

Given NHANES's complex sampling design, we analyzed the data using survey sample weights to extrapolate the results. Continuous variables were reported as means ± standard deviations or as median values with interquartile ranges. We categorized the dietary intake of Omega‐3 into three groups based on tertiles and compared differences using either a t‐test or a Kruskal–Wallis test. For categorical variables, we expressed proportions and employed a chi‐square test to evaluate differences. To investigate the association between Omega‐3 and serum testosterone levels in adult males, we applied multivariable regression analysis, which included an unadjusted model (Model I), a minimally adjusted model (Model II, adjusted for age and race), and a fully adjusted model (Model III, adjusted for age, race, BMI, diabetes, hypertension, and high cholesterol). Because the distribution of serum testosterone was skewed, a natural logarithmic transformation was performed (Han et al. 2021; Qin et al. 2022; Su et al. 2025). Statistical analyses were performed by EmpowerStats software and R version 4.1.1. Statistical significance was set at *p* < 0.05.

## 3 Results

**FIGURE 1:**
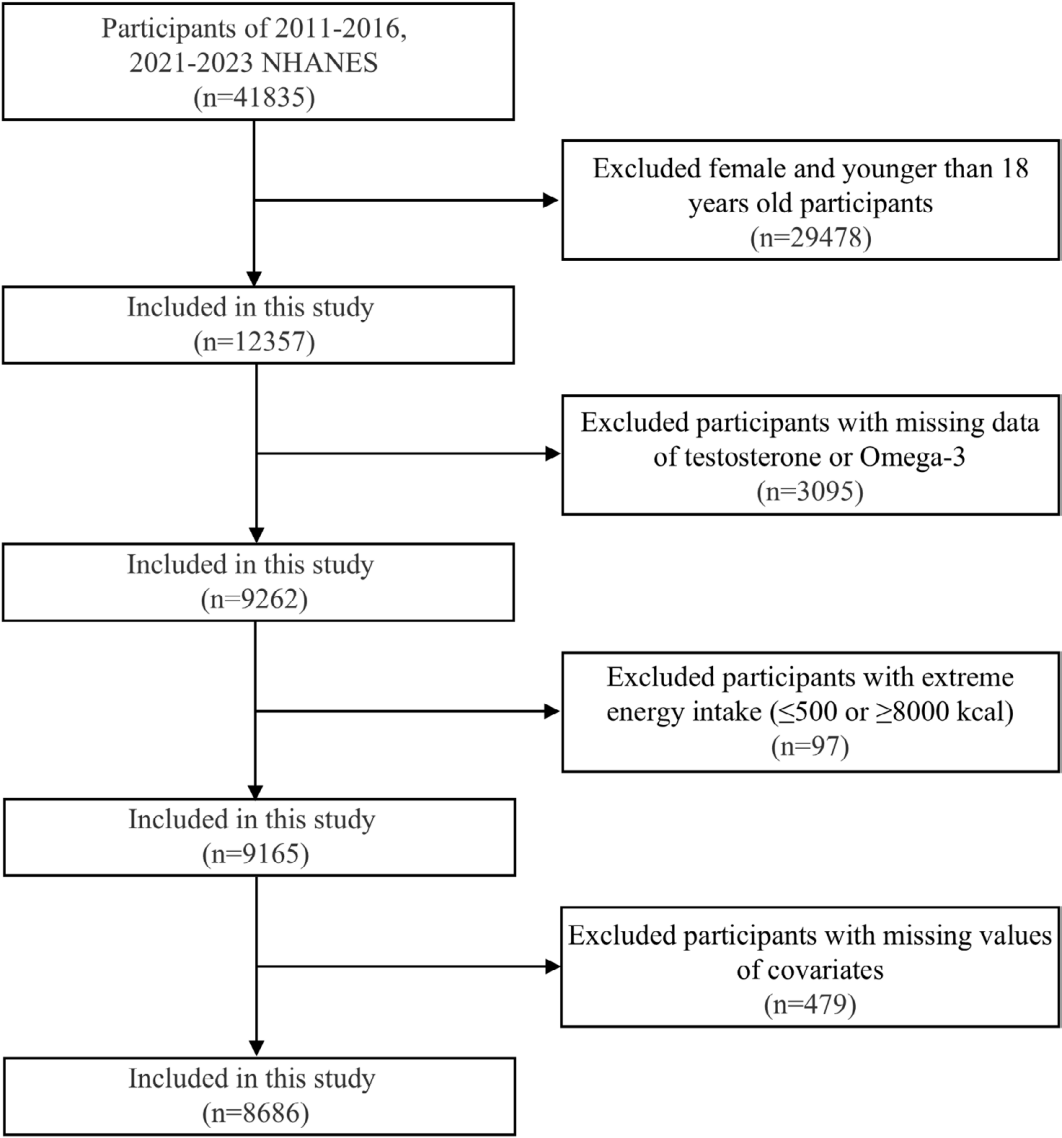
Flow chart population included in this omega‐3 and serum testosterone association analysis.

A total of 8686 participants were included in the analysis. The participant selection process is illustrated in Figure 1. The clinical characteristics of the participants sorted by dietary intake of total Omega‐3 tertiles are represented in Table 1. Among the total Omega‐3 tertiles, there were significant differences in baseline data on age, race, and hypertension (*p* < 0.001), with no significant differences in baseline data on diabetes, testosterone, BMI, and high cholesterol. Serum testosterone levels showed no significant differences among the three Omega‐3 groups (*p* = 0.958).

**TABLE 1:** Baseline characteristics of participants with serum testosterone.

Total omega‐3 fatty acids	Low	Middle	High	*p*
Number of subjects	2897	2893	2896	
Age (years)	51 (34)	49 (31)	47 (30)	< 0.001
Body mass index (kg/m^2^)	27.6 (7.3)	27.6 (7.2)	27.85 (7.3)	0.705
Testosterone (ng/dL)	402 (231)	398 (231.38)	404.64 (228.47)	0.958
Body mass index categorica				0.251
< 25 (kg/m^2^)	875 (30.20%)	803 (27.76%)	835 (28.83%)	
25–30 (kg/m^2^)	1027 (35.45%)	1096 (37.88%)	1068 (36.88%)	
≥ 30 (kg/m^2^)	995 (34.35%)	994 (34.36%)	993 (34.29%)	
Race				< 0.001
Mexican American	307 (10.60%)	406 (14.03%)	380 (13.12%)	
Other Hispanic	284 (9.80%)	290 (10.02%)	277 (9.56%)	
Non‐Hispanic White	1272 (43.91%)	1276 (44.11%)	1331 (45.96%)	
Non‐Hispanic Black	599 (20.68%)	505 (17.46%)	513 (17.71%)	
Other Race—Including Multi‐Racial	435 (15.02%)	416 (14.38%)	395 (13.64%)	
Hypertension				< 0.001
Yes	1116 (38.52%)	954 (32.98%)	939 (32.42%)	
No	1781 (61.48%)	1939 (67.02%)	1957 (67.58%)	
Diabetes				0.169
Yes	432 (14.91%)	409 (14.14%)	382 (13.19%)	
No	2465 (85.09%)	2484 (85.86%)	2514 (86.81%)	
High cholesterol				0.176
Yes	1039 (35.86%)	1085 (37.50%)	1020 (35.22%)	
No	1858 (64.14%)	1808 (62.50%)	1876 (64.78%)	

*Note:* Data are medians (interquartile ranges) or number of subjects (percentage).

Tables 2 and 3 show the regression results for serum testosterone levels and dietary Omega‐3 intake. DPA exhibited a significant positive association with serum testosterone (*β* = 0.29, 95% CI: 0.09 to 0.49, *p* = 0.004) in the non‐adjusted model, which remained significant after adjustment (Model II: *β* = 0.23, 95% CI: 0.03 to 0.43, *p* = 0.024; Model III: *β* = 0.2, 95% CI: 0.01 to 0.38, *p* = 0.041). Other fatty acids (the total Omega‐3, ALA, SDA, EPA, and DHA) did not demonstrate statistical significance across all models. After dividing the fatty acid into three equal parts, in the non‐adjusted model, EPA demonstrated a significant positive association with serum testosterone in the high tertile (*β* = 0.03, 95% CI: 0.01 to 0.06, *p* = 0.017), while no significant association was observed in Model II and Model III. For all other fatty acids analyzed, including total Omega‐3, ALA, SDA, DPA, and DHA, none of the tertiles demonstrated significant associations across any of the models.

**TABLE 2:** Association between omega‐3 and serum testosterone in adult males.

Exposure	Model I	Model II	Model III
Total Omega‐3	0.01 (−0.01, 0.01) 0.061	0.01 (−0.01, 0.01) 0.13	0.01 (−0.01, 0.01) 0.134
ALA	0.01 (−0.00, 0.01) 0.158	0.01 (−0.01, 0.01) 0.297	0.01 (−0.01, 0.01) 0.209
SDA	0.13 (−0.13, 0.4) 0.319	0.06 (−0.2, 0.32) 0.656	0.02 (−0.22, 0.27) 0.86
EPA	0.07 (−0.01, 0.15) 0.072	0.07 (−0.01, 0.15) 0.058	0.04 (−0.03, 0.11) 0.277
DPA	0.29 (0.09, 0.49) 0.004	0.23 (0.03, 0.43) 0.024	0.2 (0.01, 0.38) 0.041
DHA	0.05 (−0.01, 0.1) 0.057	0.05 (0.01, 0.1) 0.045	0.03 (−0.02, 0.07) 0.299

*Note:* Data are *β* (95% CI) and *p* value. Model I: adjust for none. Model II: adjust for age and race. Model III: adjust for age, race, body mass index, diabetes, hypertension, and high cholesterol.

Abbreviations: ALA, alpha‐linolenic acid; DHA, docosahexaenoic acid; DPA, docosapentaenoic acid; EPA, eicosapentaenoic acid; Omega‐3, Omega‐3 fatty acids; SDA, stearidonic acid.

**TABLE 3:** Association between omega‐3 tertile and serum testosterone in adult males.

Exposure	Model I	Model II	Model III
Total omega‐3 tertile			
Low	Reference	Reference	Reference
Middle	0.01 (−0.01, 0.04) 0.369	0.01 (−0.02, 0.04) 0.453	< 0.01 (−0.02, 0.03) 0.854
High	0.02 (−0.01, 0.05) 0.185	0.01 (−0.02, 0.04) 0.568	0.01 (−0.02, 0.03) 0.594
ALA tertile			
Low	Reference	Reference	Reference
Middle	0.03 (< −0.01, 0.05) 0.073	0.03 (< −0.01, 0.05) 0.072	0.02 (−0.01, 0.04) 0.173
High	0.01 (−0.02, 0.03) 0.667	< −0.01 (−0.03, 0.02) 0.792	< 0.01 (−0.02, 0.03) 0.936
SDA tertile			
Low	Reference	Reference	Reference
Middle	< 0.01 (−0.03, 0.03) 0.906	−0.01 (−0.04, 0.02) 0.526	−0.01 (−0.04, 0.01) 0.367
High	< 0.01 (−0.03, 0.03) 0.801	−0.02 (−0.04, 0.01) 0.269	< −0.01 (−0.03, 0.02) 0.74
EPA tertile			
Low	Reference	Reference	Reference
Middle	0.01 (−0.02, 0.04) 0.395	< 0.01 (−0.02, 0.03) 0.806	< 0.01 (−0.02, 0.03) 0.775
High	0.03 (0.01, 0.06) 0.017	0.02 (−0.01, 0.04) 0.275	0.02 (−0.01, 0.04) 0.172
DPA tertile			
Low	Reference	Reference	Reference
Middle	−0.02 (−0.05, 0.01) 0.169	−0.03 (−0.05, < 0.01) 0.057	−0.01 (−0.04, 0.01) 0.372
High	0.01 (−0.01, 0.04) 0.335	−0.01 (−0.03, 0.02) 0.606	0.01 (−0.01, 0.04) 0.25
DHA tertile			
Low	Reference	Reference	Reference
Middle	0.01 (−0.02, 0.03) 0.658	< −0.01 (−0.03, 0.02) 0.833	< −0.01 (−0.02, 0.02) 0.999
High	−0.01 (−0.04, 0.01) 0.318	−0.01 (−0.04, 0.01) 0.333	−0.01 (−0.04, 0.01) 0.35

*Note:* Data are *β* (95% CI) and *p* value. Model I: adjust for none. Model II: adjust for age and race. Model III: adjust for age, race, body mass index, diabetes, hypertension, and high cholesterol.

Abbreviations: ALA, alpha‐linolenic acid; DHA, docosahexaenoic acid; DPA, docosapentaenoic acid; EPA, eicosapentaenoic acid; Omega‐3, Omega‐3 fatty acids; SDA, stearidonic acid.

**FIGURE 2:**
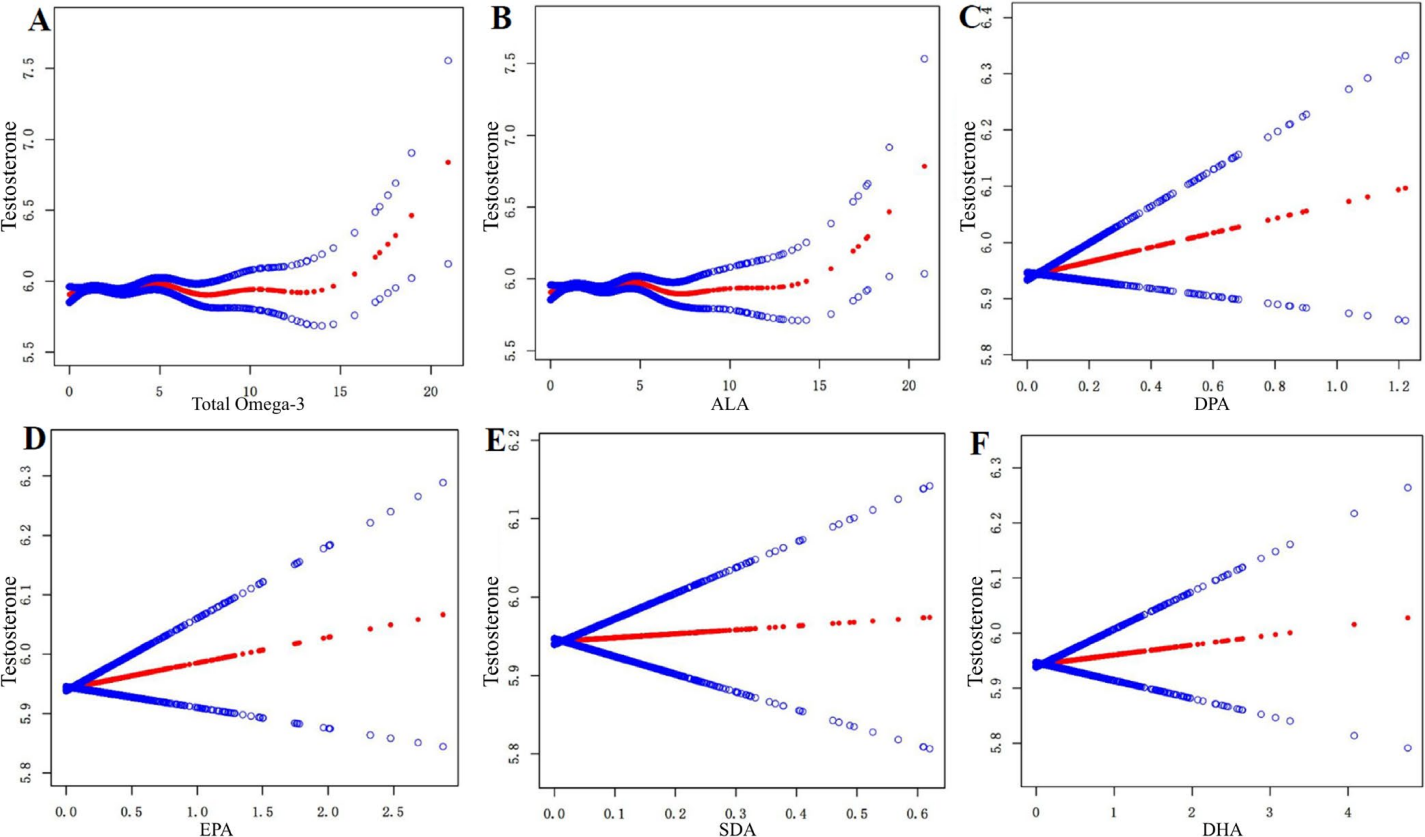
Linear and non‐linear relationship between omega‐3 and serum testosterone among adult men (A–F). (A) Testosterone and total omega‐3; (B) Testosterone and alpha‐linolenic acid; (C) Testosterone and docosapentaenoic acid; (D) Testosterone and eicosapentaenoic acid; (E) Testosterone and stearidonic acid; (F) Testosterone and docosahexaenoic acid. Generalized‐linear models adjust for age, race, body mass index, diabetes, hypertension, and high cholesterol.

We also applied generalized additive models and smooth curve fittings to evaluate the associations between Omega‐3 and serum testosterone levels in adult men (Figure 2). In adult males, EPA, DPA, DHA, and SDA showed a positive linear association with serum testosterone levels, whereas total Omega‐3 and ALA exhibited a positive but non‐linear relationship with serum testosterone.

Table 4 and Tables S1–3 show the results stratified by BMI. In the BMI < 25 kg/m^2^ group, total Omega‐3 showed a significant positive association with serum testosterone (*β* = 0.01, 95% CI: 0.01 to 0.02, *p* = 0.011). In subgroups classified by Omega‐3 type, only ALA showed a positive association (*β* = 0.01, 95% CI: 0.01 to 0.02, *p* = 0.016). After dividing the fatty acid into three equal parts (Table S1), only DPA showed a significant positive association in the high tertile (*β* = 0.06, 95% CI: 0.01 to 0.1, *p* = 0.014), while DHA exhibited a marginally significant negative association in the high tertile (*β* = −0.05, 95% CI: −0.09 to < −0.01, *p* = 0.045). In the BMI 25–30 kg/m^2^ group, only SDA showed a significant negative association (*β* = −0.44, 95% CI: −0.79 to −0.09, *p* = 0.014). After dividing the total Omega‐3 into three equal parts (Table S2), SDA still showed a significant negative association (*β* = −0.01, 95% CI: −0.1 to −0.02, *p* = 0.009), while total Omega‐3 exhibited a marginally significant negative association in the high tertile (*β* = −0.04, 95% CI: −0.08 to < −0.01, *p* = 0.045). In the BMI ≥ 30 kg/m^2^ group, significant positive associations were observed for SDA (*β* = 0.86, 95% CI: 0.28 to 1.45, *p* = 0.004), EPA (*β* = 0.24, 95% CI: 0.08 to 0.4, *p* = 0.003), DPA (*β* = 0.58, 95% CI: 0.11 to 1.05, *p* = 0.016), and DHA (*β* = 0.23, 95% CI: 0.11 to 0.35, *p* = 0.001). After dividing the Omega‐3 into three equal parts (Table S3), no significant results were observed.

**TABLE 4:** The subgroup analysis of the relationship between Omega‐3 and serum testosterone stratified by BMI.

	BMI < 25 kg/m^2^ (*N* = 2513)	BMI 25–30 kg/m^2^ (*N* = 3191)	BMI ≥ 30 kg/m^2^ (*N* = 2982)
Total omega‐3	0.01 (0.01, 0.02) 0.011	−0.01 (−0.02,0.01) 0.367	0.01 (−0.01, 0.02) 0.443
ALA	0.01 (0.01, 0.02) 0.016	−0.01 (−0.01, 0.01) 0.666	0.01 (−0.01, 0.01) 0.974
SDA	0.15 (−0.24, 0.54) 0.446	−0.44 (−0.79, −0.09) 0.014	0.86 (0.28, 1.45) 0.004
EPA	0.07 (−0.04, 0.17) 0.232	−0.1 (−0.21, 0.01) 0.08	0.24 (0.08, 0.4) 0.003
DPA	0.11 (−0.16, 0.38) 0.422	0.06 (−0.22, 0.34) 0.698	0.58 (0.11, 1.05) 0.016
DHA	0.03 (−0.04, 0.1) 0.461	−0.06 (−0.13, 0.01) 0.083	0.23 (0.11, 0.35) 0.001

*Note:* Data are *β* (95% CI) and *p* value. The model adjusted for race, age, diabetes, hypertension, and high cholesterol.

Abbreviations: ALA, alpha‐linolenic acid; BMI, body mass index; DHA, docosahexaenoic acid; DPA, docosapentaenoic acid; EPA, eicosapentaenoic acid; Omega‐3, Omega‐3 fatty acids; SDA, stearidonic acid.

## 4 Discussion

Our study, which included 8686 adult men, demonstrated that Omega‐3 intake may be positively associated with serum testosterone in adult men, potentially influenced by the type of Omega‐3 as well as BMI. Based on multivariable‐adjusted models, only dietary DPA intake showed a statistically significant positive association with serum testosterone levels in adult males. Subgroup analyses revealed a significant positive association between total Omega‐3 and ALA intake and serum testosterone in normal weight participants (BMI < 25 kg/m^2^). In the overweight subgroup (BMI 25–30 kg/m^2^), SDA exhibited a significant negative association with serum testosterone. In the obese participants (BMI ≥ 30 kg/m^2^), SDA, EPA, DPA, and DHA showed significant positive associations with testosterone levels. Considering the favorable safety profile and accessibility of Omega‐3 dietary supplementation, our results may offer preliminary evidence to guide future research on targeted nutritional strategies to support testosterone regulation in men.

To our knowledge, studies investigating the effects of Omega‐3 interventions on testosterone levels in men remain limited and show inconsistent findings. A secondary analysis of a double‐blind, parallel, placebo‐controlled, randomized controlled trial indicated that changes in testosterone levels in men are positively correlated with changes in EPA and DHA, and DHA‐rich fish oil supplements may increase testosterone levels in overweight and obese men (Abbott et al. 2020). The results of this study are similar to ours, in which EPA and DHA intake were positively associated with serum testosterone levels in obese male patients with BMI ≥ 30 kg/m^2^. However, compared to our study, the sample size included in this study was too small, with only 22 male participants. In addition, a cross‐sectional study conducted among elderly Japanese men found that higher total fish intake was associated with higher serum testosterone levels (Ito et al. 2024). Given that fish are rich in Omega‐3, this finding may be consistent with our results. Another cross‐sectional study found that self‐reported use of Omega‐3‐rich fish oil supplements was positively associated with free testosterone (Jensen et al. 2020). However, several studies have reported that Omega‐3 supplementation has no significant effect on serum testosterone levels (Giltay et al. 2012; Hughes et al. 1990). Inconsistent results of these studies may be related to the type of Omega‐3 and obesity status. Our large‐sample cross‐sectional study revealed similar findings, with a specific Omega‐3 type (DPA) showing a positive correlation with testosterone levels. This may provide a potential perspective for clinical research on Omega‐3 intake and male testosterone.

Notably, according to projections from the World Obesity Atlas 2025, nearly 3 billion adults could face overweight or obesity by 2035, placing a substantial burden on global healthcare systems. Therefore, this study conducted a subgroup analysis based on BMI to further investigate the association between Omega‐3 and serum testosterone levels in adult males. In normal weight participants (BMI < 25 kg/m^2^), total Omega‐3 and ALA exhibited a significant positive association with serum testosterone. This finding may be primarily influenced by ALA, as it constitutes the predominant component of daily Omega‐3 intake. ALA is primarily sourced from plant‐based sources such as flaxseed and soybeans (Ali et al. 2024). Therefore, in individuals with normal weight, ALA intake may indicate dietary patterns favoring green plants or vegetables, thereby promoting overall health. This offers a possible explanation for our findings. In the overweight subgroup (BMI 25–30 kg/m^2^), SDA exhibited a significant negative association with serum testosterone. It should be noted that few food sources are rich in SDA, which is not a major component of the human diet (Shahidi and Ambigaipalan 2018). Recently, plant seed oils and herbs have been widely explored as a source of SDA (Saini et al. 2021). Therefore, the result of the SDA in the overweight subgroup may be attributable to the presence of vegetarians or weight‐loss individuals in this population, which could potentially influence testosterone metabolism. Additionally, in the obese population with a BMI ≥ 30 kg/m^2^, the intake of SDA, EPA, DPA, and DHA was positively associated with serum testosterone levels. Obesity is associated with chronic low‐grade inflammation, and levels of pro‐inflammatory cytokines (such as tumor necrosis factor alpha and interleukin‐6) are typically higher in obese individuals (Shaikh et al. 2024). Our previous research indicated that inflammatory states may be negatively correlated with serum male testosterone metabolism (Su et al. 2025). Inflammatory factors may inhibit testosterone production by interfering with the normal function of the hypothalamic–pituitary–gonadal (HPG) axis (Clarke et al. 2015; Noce et al. 2020). Omega‐3 has been widely studied for its significant anti‐inflammatory capacity and modulation of inflammatory responses (Li et al. 2024; Raphael and Sordillo 2013). Therefore, it is possible that Omega‐3 may increase serum testosterone levels by ameliorating chronic inflammation associated with obesity. However, as a precursor for the biosynthesis of long‐chain fatty acids such as EPA and DHA, the conversion efficiency of ALA to EPA and DHA is relatively low, typically below 10% (Goyens et al. 2005). Therefore, the direct anti‐inflammatory effect of ALA may be limited (Burdge et al. 2002). Animal studies have also reported that ALA exhibits significantly weaker anti‐inflammatory effects in obese mice compared to EPA and DHA (Smorenburg et al. 2025). Therefore, in populations with obesity that may be accompanied by chronic inflammation, the promoting effect of ALA on serum testosterone levels may not be as pronounced. Finally, after dividing Omega‐3 into three equal parts for analysis, the results showed inconsistency with the primary analysis or marginally significant associations. One possible reason is that the effect size of the association between Omega‐3 dietary supplements and male testosterone is limited. Another possible reason is that Omega‐3 dietary supplementation lacks the recommended intake threshold associated with testosterone. Therefore, we grouped participants based on data distribution, but this may not fully align with clinical practice.

The mechanism of the association between testosterone and Omega‐3 is not fully understood. Several animal studies have explored the potential mechanisms by which Omega‐3 affects testosterone metabolism. An early study suggests that Omega‐3‐rich dietary fish oil affects testosterone synthesis and alters fatty acid composition in rat testicular plasma membranes (Sebokova et al. 1990). In a similar animal study, it was further shown that the distribution of EPA‐containing phosphatidylcholine in the testicular interstitium was well characterized, suggesting that EPA is involved in testosterone metabolism (Zaima et al. 2016). In addition, Omega‐3 can enhance the activity of steroidogenic enzymes, such as 3β‐hydroxysteroid dehydrogenase (3β‐HSD) and 17β‐hydroxysteroid dehydrogenase (17*β*‐HSD), thereby promoting testosterone biosynthesis (Odetayo and Olayaki 2023). Another potential mechanism is that chronic inflammation is an important cause of testosterone decline (Vodo et al. 2013). Several negative correlations between testosterone and inflammatory cytokines have been reported (Grandys et al. 2021; Li et al. 2022). A growing body of evidence from human and animal studies suggests that Omega‐3, primarily EPA and DHA, may inhibit inflammation (Fritsche 2006; Zhang and Spite 2012). The study shows that Omega‐3 prevents inflammation and metabolic disorders by inhibiting NLRP3 inflammatory vesicles (Yan et al. 2013). Moreover, insulin resistance has been shown to be associated with low testosterone levels (Pitteloud et al. 2005), and Omega‐3 can improve insulin sensitivity (Talukdar et al. 2011), which may consequently elevate testosterone levels. Furthermore, increased oxidative stress leads to inflammation, which in turn may affect testosterone synthesis. Research suggests that Omega‐3 may be beneficial in mitigating testicular damage by reducing germ cell apoptosis and lowering oxidative stress levels (Uygur et al. 2014). In summary, Omega‐3 can regulate testosterone metabolism through multiple pathways, including enhancing the activity of steroidogenic enzymes, inhibiting chronic inflammation, reducing oxidative stress, and improving lipid metabolism.

This study has several strengths. First, this study has the largest sample size available to study the relationship between dietary intake of Omega‐3 and testosterone. Secondly, we investigated the associations between serum testosterone and various Omega‐3s separately, and performed subgroup analyses stratified by BMI. Finally, we utilized complex sampling weights in our data analysis to enhance the external validity of the results. Nevertheless, the limitations of our study should not be overlooked. Firstly, the cross‐sectional design can only capture relationships at a specific point in time and cannot establish a causal or temporal association between dietary omega‐3 intake and testosterone levels in adult males. Therefore, our findings require validation through interventional studies employing randomized, double‐blind, placebo‐controlled trials. Secondly, due to the limited availability of data, we were unable to adjust for all potential confounding covariates, such as physical activity, smoking status, alcohol use, and total fat intake. This suggests that our findings may not fully account for the influence of other factors. Additionally, Omega‐3 supplements are important for assessing the relationship between Omega‐3 and male serum testosterone, but specific classification data for Omega‐3 supplements are not available in the NHANES database. Thirdly, Omega‐3 intake data were obtained from the dietary component of NHANES, which utilizes 24‐h dietary recall interviews. Therefore, the possibility of recall bias cannot be denied. Lastly, we could not definitively exclude participants with hypogonadism, as the necessary data were unavailable in the NHANES database.

## 5 Conclusion

Our findings indicated that Omega‐3 intake may be positively associated with serum testosterone levels in adult men, with this association potentially depending on the specific Omega‐3 type and BMI categories. Furthermore, given the nature of a cross‐sectional study, causal relationships cannot be established. Our results require validation through interventional studies. However, these results may offer preliminary insight into the potential role of specific Omega‐3 in modulating testosterone levels and support the rationale for further longitudinal and interventional research in this area.

## Author Contributions

**Liang Su:** conceptualization (equal), formal analysis (lead), supervision (equal), writing – original draft (equal), writing – review and editing (lead). **Si‐zheng Zhang:** formal analysis (equal), writing – original draft (equal), writing – review and editing (equal). **Hao‐yang Cheng:** data curation (equal), writing – review and editing (equal). **Qi Zhang:** data curation (equal), writing – review and editing (equal). **Zheng‐yang Zhou:** writing – review and editing (equal). **Jie Wu:** conceptualization (equal), supervision (equal). **Yong‐zheng Jiao:** conceptualization (equal), supervision (equal).

## Ethics Statement

The survey protocol was approved by the National Center for Health Statistics Ethics Review Board.

## Consent

All study participants submitted written informed consent.

## Conflicts of Interest

The authors declare no conflicts of interest.

## Supporting information


**Data S1:** Supporting Information.

## Data Availability

All data in the current analysis is publicly available on the NHANES website.
