# Supplementary material for: Association Between Dietary Intake of Omega‐3 Fatty Acids and Serum Testosterone in Adult Males: Results From NHANES 2011–2016 and 2021–2023
Source: Food Sci Nutr. 2025 Oct 8;13(10):e71062. doi: 10.1002/fsn3.71062 (PMC12507726; doi:10.1002/fsn3.71062)
Supplement: Supplementary file 1 — Data S1: Supporting Information. [file FSN3-13-e71062-s001.docx]

**Supplementary Material**

Supplemental Table 1. Association between Omega-3 and male serum testosterone in body mass index <25 kg/m^2^ group (N=2513)

| Exposure | Total Omega-3 | ALA | SDA | EPA | DPA | DHA |
| --- | --- | --- | --- | --- | --- | --- |
| Tertile |  |  |  |  |  |  |
| Low | Reference | Reference | Reference | Reference | Reference | Reference |
| Middle | 0.03  (-0.02, 0.07) 0.248 | 0.01  (-0.04, 0.05) 0.768 | 0.02  (-0.02, 0.07) 0.309 | 0.01  (-0.03, 0.06) 0.561 | <0.01  (-0.04, 0.05) 0.83 | -0.03  (-0.07, 0.01) 0.173 |
| High | 0.04  (-0.01, 0.08) 0.107 | 0.03  (-0.02, 0.07) 0.204 | 0.03  (-0.02, 0.08) 0.223 | 0.02  (-0.03, 0.06) 0.455 | 0.06  (0.01, 0.1) 0.014 | -0.05  (-0.09, <-0.01) 0.045 |

Data are β (95% CI) and p value. The model adjusted for age, race, diabetes, hypertension and high cholesterol. Omega-3: Omega-3 fatty acids, DHA: docosahexaenoic acid, EPA: eicosapentaenoic acid, ALA: alpha-linolenic acid, DPA: docosapentaenoic acid, SDA: stearidonic acid.

Supplemental Table 2. Association between Omega-3 and male serum testosterone in body mass index 25-30 kg/m^2^ group (N=3191)

| Exposure | Total Omega-3 | ALA | SDA | EPA | DPA | DHA |
| --- | --- | --- | --- | --- | --- | --- |
| Tertile |  |  |  |  |  |  |
| Low | Reference | Reference | Reference | Reference | Reference | Reference |
| Middle | -0.01  (-0.05, 0.03) 0.592 | -0.02  (-0.06, 0.03) 0.445 | -0.01  (-0.05, 0.03) 0.765 | 0.03  (-0.01, 0.07) 0.181 | <0.01  (-0.04, 0.04) 0.914 | -0.01  (-0.05, 0.03) 0.745 |
| High | -0.04  (-0.08, <-0.01) 0.045 | -0.04  (-0.08, <0.01) 0.072 | -0.06  (-0.1, -0.02) 0.009 | -0.01  (-0.05, 0.03) 0.706 | -0.02  (-0.06, 0.02) 0.422 | -0.04  (-0.08, <0.01) 0.062 |

Data are β (95% CI) and p value. The model adjusted for age, race, diabetes, hypertension and high cholesterol. Omega-3: Omega-3 fatty acids, DHA: docosahexaenoic acid, EPA: eicosapentaenoic acid, ALA: alpha-linolenic acid, DPA: docosapentaenoic acid, SDA: stearidonic acid.

Supplemental Table 3. Association between Omega-3 and male serum testosterone in body mass index ≥30 kg/m^2^ group (N=2982)

| Exposure | Total Omega-3 | ALA | SDA | EPA | DPA | DHA |
| --- | --- | --- | --- | --- | --- | --- |
| Tertile |  |  |  |  |  |  |
| Low | Reference | Reference | Reference | Reference | Reference | Reference |
| Middle | 0.01  (-0.04, 0.06) 0.714 | 0.04  (-0.01, 0.09) 0.147 | -0.04  (-0.09, <0.01) 0.078 | -0.04  (-0.09, 0.01) 0.093 | -0.05  (-0.09, <0.01) 0.065 | 0.03  (-0.02, 0.07) 0.268 |
| High | 0.03  (-0.02, 0.08) 0.276 | <0.01  (-0.04, 0.05) 0.877 | 0.01  (-0.04, 0.06) 0.692 | 0.03  (-0.02, 0.08) 0.242 | 0.01  (-0.04, 0.06) 0.697 | 0.03  (-0.02, 0.08) 0.178 |

Data are β (95% CI) and p value. The model adjusted for age, race, diabetes, hypertension and high cholesterol. Omega-3: Omega-3 fatty acids, DHA: docosahexaenoic acid, EPA: eicosapentaenoic acid, ALA: alpha-linolenic acid, DPA: docosapentaenoic acid, SDA: stearidonic acid.
